# Ammoniated Tr. of Gum Guaiac in the Treatment of Females

**Published:** 1901-01

**Authors:** N. F. Howard

**Affiliations:** Dahlonega, Ga.


### AMMONIATED TR. OF GUM GUAIAC IN THE TREATMENT OF FEMALES.

BY N. F. HOWARD, M.D.,

DAHLONEGA, GA.

Nine years ago I had a severe attack of pharyngitis when absent from home in North Carolina. This attack was relieved in a few days by quinine, calomel, and the application of turpentine poultices to my throat and neck, and a gargle of chloride of potash and carbolic acid, yet I was confined to the house eight or ten days. Three years ago last March I had another attack of the same disease of equal severity, also attended with a cough.

Dr. H. C. Whelchel was called in to attend me, and in a few days the severity of the case had passed by and I was very soon up and about. During this spell Dr. Whelchel gave me a cough preparation, to which was added the ammoniated tincture of gum guaiac. At once I made the inquiry of the doctor, "Did you give this guaiac for the cough or the inflamed membrane of the pharynx?" "For both," he replied, "but especially for the inflammation of the throat."

In reply I said to him: "This is Dr. Dewees's preparation for female troubles—suppression of the menses, from cold, which I have been using for more than forty years in such cases. Yet I did not know that the same preparation of guaiac was in use for throat troubles." I wish to say at this point that I have called attention to this special case in myself for the purpose of making the statement and calling attention to the fact, which is well known to the profession, that Prof. Dewees, in his work upon diseases of females, uses the formula of gum guaiac, aromatic spirits of ammonia, allspice and soda, in preparing his compound tincture of gum guaiac. This is the preparation that he recommended to be used in rheumatism, and kindred diseases, and especially with women who had, from cold or other causes, brought about menstrual disarrangements and entire suppression of the same. Dr. Dewees's work was used by myself as a text-book. I remember very well that his formula and recommendation was lightly spoken of, even by some leading medical men, and I have heard him called by some, "Old Granny Dewees." Yet, I decided to accept his formula of this medicine in my treatment of rheumatism, neuralgias, and especially female trouble which had originated from cold or any other imprudence, and suppression of the menses. I am glad to say at this time that I never have been disappointed, to any great degree, in all the years of my practice in the use of this medicine. I do not remember a single instance in the many cases in which I used it, for nearly fifty years now, in which it failed to accomplish a relief and recovery in all such cases, except in such cases as have been complicated with organic disease. It is true that other medicines, as adjuvants, it is proper to say, often should be used to aid in this treatment. My plan has been for the patient to use one drachm, night and morning, in a little sweetened ginger tea, taken from one monthly period to the next. At which time, or a day or two before the period is expected, I found that the medicine called tincture of antacrida, which was a favorite of Prof. Fenner of New Orleans, gave great comfort during the menstrual period, taken twenty drops, night and morning, in a cup of sweetened ginger tea. As soon as comfort is afforded, or the menses appear, the antacrida is discontinued, and then from this period until the next, use again as before, the ammoniated tincture of gum guaiac. If the patient is chlorotic, some preparation of iron and nux vomica should be given her; also small doses of tincture of digitalis, or any other tonic the attending physician may select. When there is enlargement of the abdomen or constipation, a favorite pill to be given for two or three nights just before the period is expected, is aloes and extract of nux vomica. I find that this preparation of ammoniated tincture of gum guaiac, as prepared by Dr. Dewees, is more efficient in the treatment of rheumatism and neuralgia than any formula of the tincture of gum guaiac. I wish to state that it has been found at our house, by Mrs. J. A. Howard, that sugar, when the tincture is mixed with a little water, covers the bitter taste of the guaiac, and that it is so palatable that even children will take it without complaint, and in cases of hoarseness, cough, croup, or any form of sore throat, children large enough to do so, will call for it from time to time, when suffering.

Permit me to add one more fact. Add twenty drops of ammoniated tincture of guaiac, two teaspoonfuls of sugar to half a glass of water, place it convenient for the ants if there are any about the house, and they will go to this medicine, drink their fill of the mixture, and then fall into it and die, so that in a few days all the ants will disappear from about the house.

